# Ruxolitinib in adult dermatomyositis with anti-TIF1γ antibody: a case report and literature review

**DOI:** 10.3389/fimmu.2025.1591631

**Published:** 2025-06-12

**Authors:** Xu Jing, Wang Min, Liu Xia, Li Shanshan

**Affiliations:** ^1^ Department of Cardiology, State Key Laboratory of Cardiovascular Diseases, Fuwai Hospital, National Center for Cardiovascular Diseases, Chinese Academy of Medical Sciences and Peking Union Medical College, Beijing, China; ^2^ Department of Hematology, China-Japan Friendship Hospital, Beijing, China; ^3^ Department of Rheumatology, Key Laboratory of Myositis, China-Japan Friendship Hospital, Beijing, China

**Keywords:** ruxolitinib, dermatomyositis, essential thrombocytosis, JAK2 V617F mutation, anti-TIF1γ antibody

## Abstract

Ruxolitinib is a selective JAK1/2 inhibitor, and its application in treating adult-onset dermatomyositis(DM) has rarely been described. Here, we report a case of an adult-onset DM patient with anti-TIF1γ antibody, who also suffered from essential thrombocytosis related to the *JAK2 V617F* mutation. Her clinical symptoms of DM markedly improved after ruxolitinib treatment, whereas her platelet count did not significantly change. In addition, we reviewed previous studies on the treatment of adult DM with ruxolitinib, and summarized reports of the J*AK2 V617F* mutation in adult idiopathic inflammatory myopathy.

## Introduction

The Janus kinase(JAK)-signal transducer and activator of transcription (JAK-STAT) pathway plays a pivotal role in physiological functions, such as hematopoiesis and immune balance (1). JAK inhibitors are approved for treating some hematological and autoimmune diseases. For dermatomyositis(DM), increasing evidence suggests that JAK inhibitors can effectively manage interstitial lung disease(ILD) and refractory rashes (2–4). A recent Chinese retrospective study suggests the superiority of tofacitinib over calcineurin inhibitors in patients with anti-MDA5^+^DM (5). Therefore, JAK inhibitors are increasingly used to treat ILD or cutaneous symptoms despite being off-label in DM.

Ruxolitinib is a selective JAK1/2 inhibitor that has been approved for the treatment of primary and secondary myelofibrosis(MF). In addition, it is administered to treat essential thrombocytosis (ET) in clinical practice, although its efficacy remains controversial in this indication (6, 7). However, few reports focused on the simultaneous treatment of DM and ET with ruxolitinib. Here, we report a case of an adult DM with anti-TIF1γ antibody and ET with the *JAK2 V617F* mutation. In addition, we reviewed previously published cases of ruxolitinib treatment for adult-onset DM and *JAK2 V617F* mutation with adult idiopathic inflammatory myopathy(IIM) to broaden our understanding.

## Case report

In May 2022, a 34-year-old woman developed heliotrope rash, Gottron’s sign, and V sign. These rashes were itchy. Her muscle strength was normal. DM was suspected by dermatologists. She received prednisone (30mg daily) and hydroxychloroquine (HCQ)(withdrawn in August 2022 due to adverse effects), a combination therapy of methotrexate and thalidomide(discontinued in March 2023 for the second relapse), and Chinese herbal medicine(stopped in December 2023 because of personal reason). This resulted in partial improvement of skin lesions with hyperpigmentation. Her symptoms relapsed twice as the dose of prednisone was decreased to 15mg daily in December 2022 and March 2023, respectively. She had to increase prednisone to 30 mg daily. She gradually reduced the dosage of prednisone and stopped all treatments in December 2023, while her symptoms did not completely alleviate. In February 2024, the patient developed skin lesions (in addition to the aforementioned rashes, erythema also appeared on the cheeks and trunk). Laboratory tests showed normal range of muscle enzyme profiles and elevated platelet count (PLT 690×10^9^/L). Skin biopsy showed liquefaction degeneration of the basal layer and mucin deposition in the dermis. Bone marrow puncture confirmed thrombocythemia. In addition, a *JAK2 V617F* mutation was detected by quantitative real-time polymerase chain reaction, confirming the diagnosis of ET. The patient did not have family history of related hematological disorders or autoimmune diseases. She started taking aspirin 100mg daily to prevent thrombosis, and did not receive treatment for DM due to personal reason.

**Figure 1:**
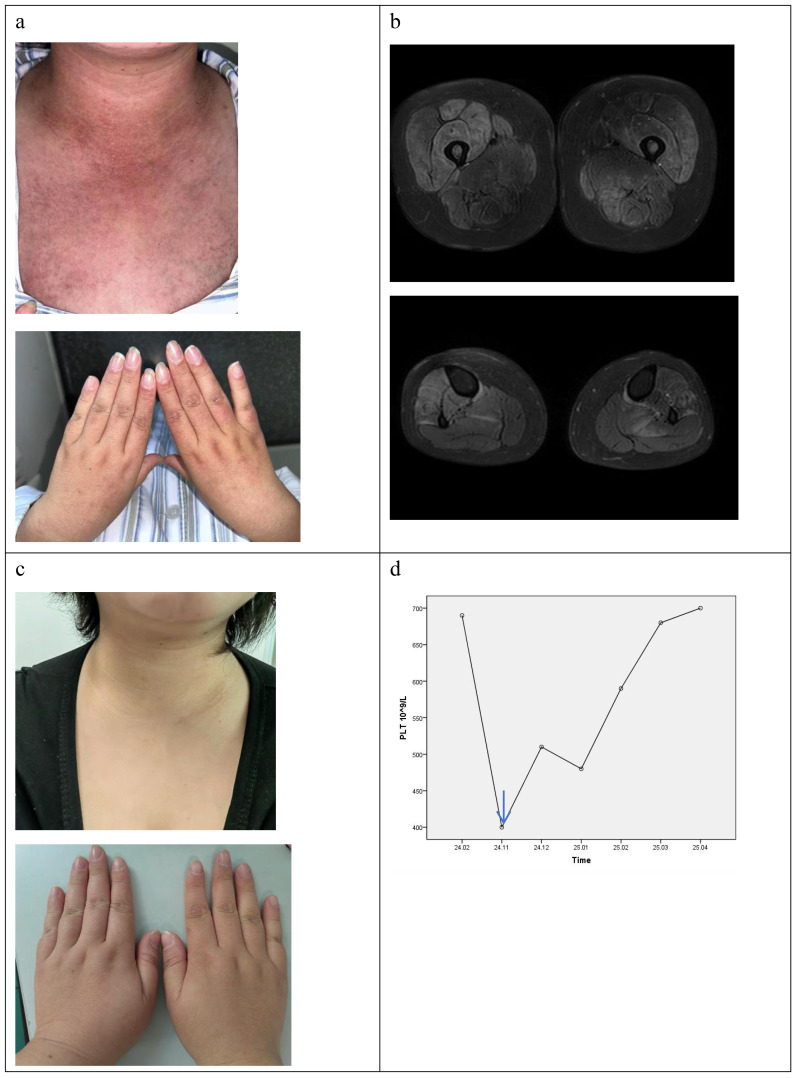
the clinical feature of patient with DM and ET. **(a)** the rash before ruxolitinib. **(b)** the T2 MRI of lower limb before ruxolitinib. **(c)** the rash after ruxolitinib at 5th month. **(d)** the change of platelet count (blue arrow: ruxolitinib start).

In November 2024, she came to our department due to mild muscle weakness and diffuse rashes, which lasted for four months. On physical examination, she had heliotrope rash, Gottron’s sign, V sign, diffuse erythema in cheeks, arms, and trunk (**Figure 1a**). She had muscle weakness with an MMT8 score of 76. Myositis-specific autoantibodies(MSA) test(immunoblotting test) showed positive result of anti-TIF1γ antibody. The level of creatine kinase (CK) was normal. Further whole-body muscle MRI showed muscle inflammation (**Figure 1b**). PET-CT imaging showed normal metabolic activity, and no significant abnormalities were observed during gastroscopy and colonoscopy. Therefore, TIF1γ**^+^** DM was diagnosed according to the 2018 ENMC classifications (8). Considering the patient with ET simultaneously, we consulted a hematologist for treatment advice. She took low dose ruxolitinib (5 mg twice a day), prednisone 30mg daily(equivalent to 0.5mg/kg) and aspirin 100mg daily. There was a significant improvement in skin lesions and a reduction in itching within 1 weeks. Her muscle strength gradually returned to normal, and her rash almost disappeared after taking ruxolitinib for 2 months. The prednisone dose was successfully reduced to 10mg daily at the last follow-up in April 2025, but there was no significant change in platelet count (**Figures 1c, d**).

## Literature review

We searched PubMed for studies on ruxolitinib in adult-onset DM patients using the keywords “ruxolitinib” and “dermatomyositis” in English from 2010 to 2024. We reviewed literature that clearly diagnosed DM and excluded juvenile DM, and identified 9 adult patients with DM (9–14). All patients were female with ages ranging from 29 to 79 years old. Among them, 5 cases were with anti-TIF1γ antibody, and 3 cases were with anti-NXP2 antibody, anti-MDA5 antibody and anti-SAE antibody, respectively. The earliest published case(no.1) did not specify the type of MSA. One of the patients was topically treated with ruxolitinib, whereas the remaining patients received oral treatment. Except for one case not described, the ruxolitinib dosage was usually between 20 to 30 mg daily. In addition, case no.1 suffered from MF secondary to polycythemia with the *JAK2 V617F* mutation. All patients’ skin lesions had improved. One patient died of cancer and organ dysfunction. The clinical data are shown in **Table 1**. In addition, a case of adult antisynthetase syndrome (ASS) with anti-PL12 antibody was treated with ruxolitinib, which improved cutaneous lesions, fever, synovitis, and maintaining stable ILD (15).

**Table 1:** Characteristics of patients and treatment response to ruxolitinib in dermatomyositis.

No.	Country	Age Gender	Disease duration	MSA	Involvement organ	Previous treatment	Ruxolitinib (mg/d)	Prognosis
1	Germany	72F	1y	NA	Skin,Muscle	GC,AZA,IVIG, MMF	30	Skin lesions completely resolved; Regain muscle strength
2	Germany	40sF	6y	Anti-TIF1r	Skin,Muscle	GC,AZA,MTX,IVIG,RTX,HCQ, CsA,MMF, CYC, etanercept	30	Skin lesions improved Muscle strength improved
3	France	62F	5m	Anti-MDA5	Skin,Muscle,RP-ILD	GC,CYC,IVIG	30	Stable, but died of cancer and organ dysfunction after 1y
4	France	59F	5y	Anti-TIF1r	Skin,Muscle	GC,HCQ,IVIG, PLEX,MTX,AZA	30	Skin lesions improved MMT8 stable
5	France	79F	4y	Anti-SAE	Skin,Muscle	GC,MTX,IVIG, MMF	30	Skin lesions improved MMT8 stable
6	France	84F	1y	Anti-TIF1r	Skin,Muscle	GC,MTX,AZA, IVIG	20	Skin lesions improved CK level decreased
7	France	29F	4y	Anti-TIF1r	Skin,Muscle	GC,HCQ,MTX, AZA,MMF,IVIG	20	Skin lesions improved MMT8 improved
8	USA	64F	28y	Anti-NXP2	Skin,Muscle	IVIG,MTX,AZA,HCQ,CsA,RTX, adalimumab,	topical	Skin lesions improved
9	France	45F	6y	Anti-TIF1r	Skin,Muscle	GC,HCQ,MTX, AZA,MMF,RTX,CYC,IVIG	NA	Skin lesions improved Muscle strength improved

AZA, azathioprin; CK, creatine kinase; CsA, cyclosporine; CYC, cyclophosphamide; GC, glucocorticoid; HCQ, hydroxychloroquine; IVIG, intravenous immune globulin; MMF, mycophenolate mofetil; MMT8, manual muscle testing 8; MSA, myositis specific antiboy; MTX, methotrexate; NA, not available; PLEX, plasma exchange; RTX, rituximab.

Next, we searched PubMed for studies on *JAK2* V617F mutation with adult IIM using the keywords “*JAK2* V617F” and “idiopathic inflammatory myopathy”(IIM) or “dermatomyositis” or “polymyositis(PM)” or “antisynthetase syndrome(ASS)” or “immune-mediated necrotizing myopathy” or “inclusion body myositis” in English from 2000 to 2024. Literature that clearly diagnosed the above disease and excluded juvenile DM was reviewed. Overall, only 3 cases of IIM combined with *JAK2* V617F mutation were reported (9, 16). The first patient was diagnosed with post-polycythemia vera MF 1 year after the onset of DM. The second patient was diagnosed with ET 6 years after the onset of ASS (anti-PL7 positive antibody). The third patient was only described to have PM combined with the *JAK2* V617F mutation (**Table 2**).

**Table 2:** the characteristics and prognosis of patients with IIM and JAK2 V617F mutation.

Published year	Country	Age/Gender	Disease duration	IIM subtype	Hematological disease	Treatment	Prognosis
2014	Germany	72F	1y	DM	post–polycythemia vera myelofibrosis	GC,AZA,IVIG,MMF, Ruxolitinib	Skin lesions completely resolved; Regain muscle strength; Platelet recovery; Spleen size decreased.
2021	China	33F	6y	ASS(PL7)	ET	GC,TAC	DM activity(CDASI) decreased; Platelets not change.
2021	China	60M	NA	Polymyositis	NA	NA	NA

ASS, antisynthetase syndrome; AZA, azathioprin; CDASI, cutaneous dermatomyositis disease area and severity index; DM, dermotomyositis; GC, glucocorticoid; IIM, idiopathic inflammatory myopathy; IVIG, intravenous immune globulin; MMF, mycophenolate mofetil; NA, not available; TAC, tacrolimus.

## Discussion

This case highlights a rare and complex manifestation of DM with anti-TIF1γ antibody and ET with *JAK2 V617F* mutation. On the one hand, ruxolitinib significantly improved the clinical symptoms of DM, providing new evidence for the efficacy of ruxolitinib in treating DM; on the other hand, the special feature of this case was that the patient had a *JAK2 V617F* mutation, which may be one of the reasons for the excellent therapeutic effect of ruxolitinib.

JAK inhibitors reduce the production of various cytokines including type I interferons(IFN-I) and suppress inflammation, which is a novel treatment option for DM in recent years. Most studies have focused on the efficacy of tofacitinib, baricitinib, and upadacitinib in the treatment of DM (2–4). Here, we reported that another JAK inhibitor, ruxolitinib, had successfully treated DM. Although it has been studied in juveniles and adults with refractory DM, few studies focused on the latter, with only several case reports. A previous study confirmed that IFN-I reproduced the main DM pathological findings, including muscle atrophy and vasculopathy, and ruxolitinib inhibited the pathogenic effects of IFN-I in both muscle and endothelial cells *in vitro (* 14). This provided evidence for the treatment of DM with ruxolitinib. In general, ruxolitinib will take effect within 3 months and can be sustained over a long-term follow-up (12). In terms of the therapeutic effect, previous literature indicated that ruxolitinib was more effective for refractory rashes than for myositis (14). In the literature review, all 9 patients demonstrated significant improvement in skin lesions after switching to ruxolitinib. However, 7 out of 9 patients showed stable or improved muscle strength, indicating that ruxolitinib may exhibit a good effect on muscle involvement.

Noteworthily, the coexistence of DM and ET with *JAK2 V617F* mutation indicated the possible complex interaction between hematologic and autoimmune diseases. The *JAK2 V617F* mutation drives the activation of the JAK-STAT pathway, which is crucial to hematopoiesis and immune regulation (17). According to reports, 50% of patients with ET have *JAK2 V617F* mutation (18). Case reports described patients harboring this gene mutation and various autoimmune diseases, such as rheumatoid arthritis, Sjögren syndrome and anti-neutrophil cytoplasmic antibody associated vasculitis (16, 19). In our patient, the *JAK2 V617F* mutation may have resulted in both hematologic and autoimmune manifestations through immune dysregulation and chronic inflammation, although the exact mechanism remains unclear. Therefore, JAK inhibitors may be a valuable option for patients who exhibit this gene mutation.

At present, there are over 10 registered studies on JAK inhibitors in the treatment of DM, among which the results of tofacitinib and baricitinib in DM had shown good effects in improving the disease (20, 21). These results further enhanced the confidence in using JAK inhibitors to treat DM. However, we should be alert to the adverse effects caused by the widespread administration of JAK inhibitors, especially when prescribed off-label in the treatment of DM. Tofacitinib has increased the risk of tumor and cardiovascular event in RA (22, 23). According to the published data in RA, surveillance demonstrated an HR of 1.48 (95% CI 1.04 to 2.09) for tofacitinib versus tumor necrosis factor inhibitors (23). However, the subsequent published meta-analysis showed that JAK inhibitors did not associate with a higher incidence of malignancy compared with placebo in RA or other autoimmune/inflammatory diseases (24). Baricitinib, another JAK1/2 inhibitor, has been more widely administered than ruxolitinib in the treatment of DM. Some adverse effects of baricitinib have been reported, such as cytomegalovirus infection and venous thrombophlebitis (3). Ruxolitinib has been approved for the treatment of some hematological malignancies; however, when administered for treating DM, the issue of adverse effects should also be addressed. For example, patient no.3 in the review section developed cancer in the second year. In addition, as anti-TIF1γ antibody is considered a tumor associated myositis antibody, the administration of ruxolitinib needs to be given extra attention (25).

## Conclusion

This patient simultaneously suffered from DM with anti-TIF1γ antibody and ET harboring *JAK2 V617F* mutation, and ruxolitinib improved the clinical symptoms of DM. The underlying association between DM and *JAK2 V617F* mutation warrants further attention and investigation.

## Data Availability

The original contributions presented in the study are included in the article/Supplementary Material. Further inquiries can be directed to the corresponding author.
